# Submarine metalliferous carbonate mounds in the Cambrian of the Baltoscandian Basin induced by vent networks and water column stratification

**DOI:** 10.1038/s41598-022-12379-y

**Published:** 2022-05-19

**Authors:** J. Javier Álvaro, Lars E. Holmer, Yanan Shen, Leonid E. Popov, Mansoureh Ghobadi Pour, Zhifei Zhang, Zhiliang Zhang, Per Ahlberg, Heikki Bauert, Laura González-Acebrón

**Affiliations:** 1 Instituto de Geociencias (CSIC-UCM), Dr. Severo Ochoa 7, 28040 Madrid, Spain; 2 Department of Earth Sciences, Palaeobiology, Uppsala University, 75236 Uppsala, Sweden; 3 State Key Laboratory of Continental Dynamics, Shaanxi Key Laboratory of Early Life and Environments, Department of Geology, Northwest University, Xi’an, 710069 China; 4 School of Earth and Space Sciences, University of Science and Technology of China, Hefei, 230026 China; 5 Department of Natural Sciences, National Museum of Wales, Cathays Park, Cardiff, CF10 3NP UK; 6 Department of Geology, Faculty of Sciences, Golestan University, Gorgan, 49138-15739 Iran; 7 Department of Geology, Lund University, Sölvegatan 12, 22362 Lund, Sweden; 8 Geological Survey of Estonia, Tartu maantee 85, 11412 Tallinn, Estonia; 9 Universidad Complutense, José Antonio Novais 2, 28040 Madrid, Spain

**Keywords:** Geochemistry, Geology, Palaeontology, Sedimentology, Environmental sciences, Solid Earth sciences

## Abstract

Two massive precipitation events of polymetallic ore deposits, encrusted by a mixture of authigenic carbonates, are documented from the Cambrian of the semi-enclosed Baltoscandian Basin. δ^34^S (‒9.33 to ‒2.08‰) and δ^33^S (‒4.75 to ‒1.06‰) values from the basal sulphide breccias, sourced from contemporaneous Pb–Zn–Fe-bearing vein stockworks, reflect sulphide derived from both microbial and abiotic sulphate reduction. Submarine metalliferous deposits were triggered by non-buoyant hydrothermal plumes: plumes of buoyant fluid were trapped by water column stratification because their buoyancy with respect to the environment reversed, fluids became heavier than their surroundings and gravitational forces brought them to a halt, spreading out laterally from originating vents and resulting in the lateral dispersion of effluents and sulphide particle settling. Subsequently, polymetallic exhalites were sealed by carbonate crusts displaying three generations of ikaite-to-aragonite palisade crystals, now recrystallized to calcite and subsidiary vaterite. *T*_h_ of fluid inclusions in early calcite crystals, ranging from 65 to 78 ºC, provide minimum entrapment temperatures for carbonate precipitation and early recrystallization. δ^13^C_carb_ (‒1.1 to + 1.6‰) and δ^18^O_carb_ (‒7.6 to ‒6.5‰) values are higher than those preserved in contemporaneous glendonite concretions (‒8.5 to ‒4.7‰ and ‒12.4 to ‒9.1‰, respectively) embedded in kerogenous shales, the latter related to thermal degradation of organic matter. Hydrothermal discharges graded from highly reduced, acidic, metalliferous, and hot (~ 150 ºC) to slightly alkaline, calcium-rich and warm (< 100 ºC), controlling the precipitation of authigenic carbonates.

## Introduction

Hydrothermal calcium carbonate precipitates occur as metre-sized tufa mounds and chimneys in Ikka Fjord, Greenland^1,2^, Mono Lake, California^3–6^ and the Lost City Hydrothermal Field, Mid-Atlantic Ridge^7,8^. Most of these carbonate structures grow as a result of mixing warm (< 100 ºC), high pH (8‒10), calcium-rich spring waters with cool, bicarbonate-rich waters^2,8,9^. The geochemistry of venting waters is primarily controlled by both low-temperature hydrothermal and serpentinization reactions in some basement rocks, which comprise syenites-carbonatites, rhyolites and peridotites-gabbros, respectively^1,6,7,10–12^. The precipitation of variable mixtures of ikaite, vaterite, aragonite and calcite was controlled by the seawater carbonate buffered system and the pH and temperature of vent fluids. The carbonate structures lack (i) metalliferous sulphide minerals due to the lack of metals and/or sulphide in the vents, and (ii) anhydrite because mixing temperatures are low enough to prevent anhydrite saturation^2,9^.

In contrast, fluids venting from hotter “black smoker” chimneys emit high temperature (300‒350 ºC), slightly acidic, metal-, sulphide- and Ca-rich fluids into cold seawater that develop seafloor precipitates of sulphate (anhydrite) and sulphides^13^. Temporal variability in vent-fluid composition, pH and temperature commonly reflect changes in the nature of the underlying heat source, contribution of magmatic fluids, residence time of fluids and the involvement of volcanic vs. sedimentary material in the hydrothermal circulation cells^14,15^. In sediment-covered ridges, basalts are both interbedded and overlain by sediments, whose aluminosilicate and organic matter content can directly control the physicochemical conditions of hydrothermal fluids^16^.

Hydrothermal structures caused by the precipitation of two contrasting phases, an early mineralization of polymetallic sulphides and sulphates overprinted by a latter precipitation of authigenic carbonates, reflecting temperature and pH gradients not attributable to changes in magmatic sources, are rare and poorly understood^13,16^. In this study, we examine micro-textural and compositional variations of two massive precipitation events of polymetallic ore breccia deposits encrusted by a mixture of authigenic carbonates in Cambrian strata from the semi-enclosed Baltoscandian Basin, on the island of Öland, Sweden. Carbonate mounds nucleating on sulphide breccias are documented, for the first time, from a Cambrian kerogenous seafloor recording contemporaneous precipitation of glendonite concretions.

## Geological and stratigraphic background

**Figure 1:**
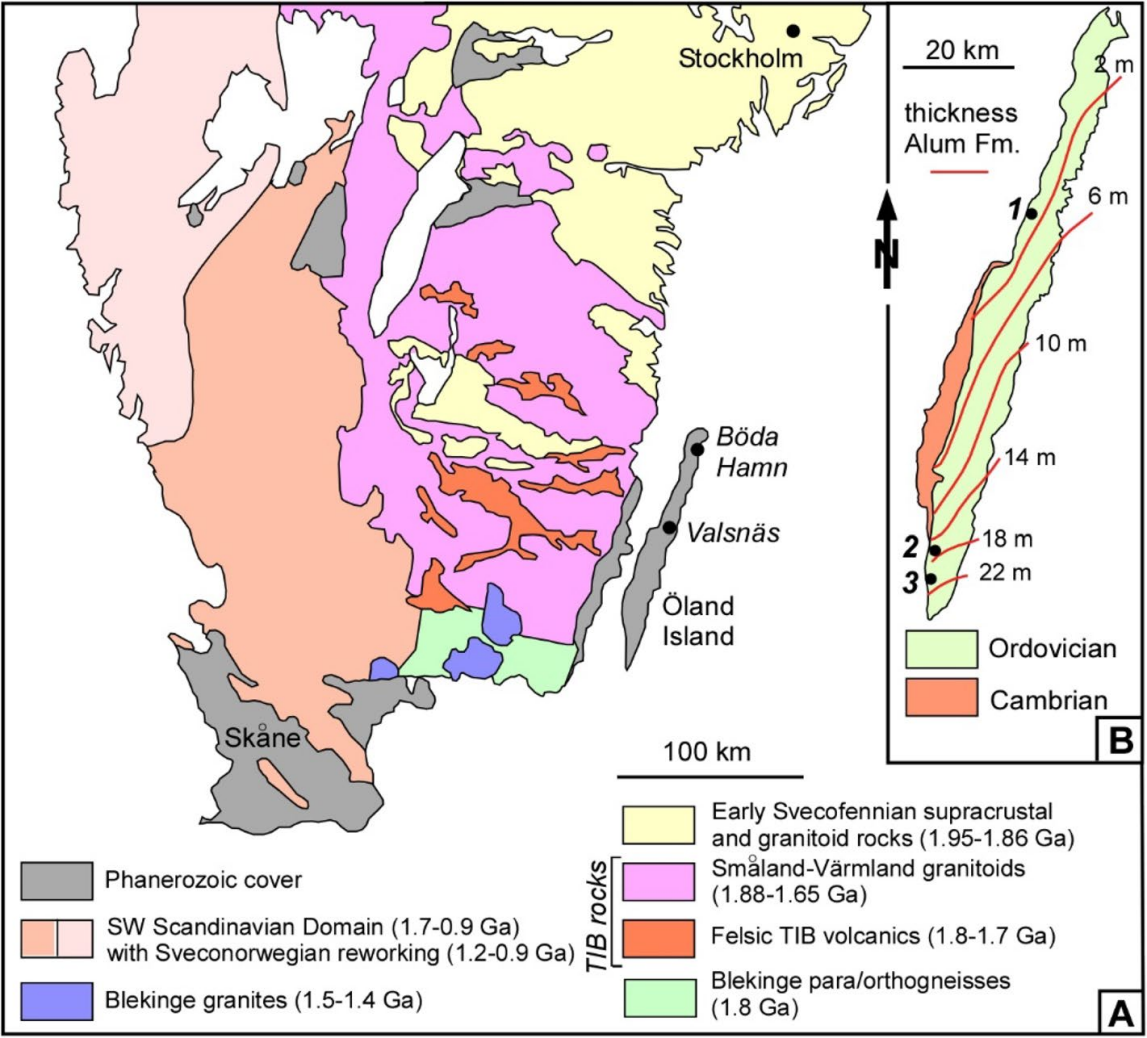
**(A)** Map of southern Sweden showing the distribution of Phanerozoic rocks and the Transscandinavian Igneous Belt (TIB)^17^. (**B)** Schematic map of Öland showing location of sections discussed in the text: 1, Bruddesta; 2, Degerhamn; 3, Grönhögen-2015 borehole. Isopaches for the Alum Shale Formation after^24^.

The Transscandinavian Igneous Belt is a giant array of large massifs with granitoid rocks and associated mafic intrusions, with ages ranging between *ca.* 1.85 and 1.67 Ga (Fig. 1A). In its SE edge, the Precambrian basement of Öland represents a lateral prolongation of the so-called Småland-Värmland Belt, and is unconformably overlain by Cambrian-Ordovician strata. It displays compositional trends ranging from ultramafic rocks (dunites and peridotites) to granites^17^. During the 1970s‒1990s, the Phanerozoic sedimentary cover of the Swedish sector was explored for its hydrocarbon potential^18^; holes drilled on Öland reached Precambrian rocks at depths ranging from 201 m at Valsnäs to 162 m at Böda Hamn (Fig. 1B). Drill core samples are 1788 ± 5 Ma and 1799 ± 4 Ma in age (U‒Pb dates on zircon), respectively. They range from quartz monzodiorites to quartz monzonites and are similar to other granitoids exposed on the continental belt^19^.

The metalliferous carbonate mounds and glendonite concretions described below lie on the Cambrian cover of the Småland-Värmland Belt. The cover comprises the fine-grained siliciclastic File Haidar and Borgholm formations (Cambrian Epoch 2 to Miaolingian in age) and the unconformably overlying Alum Shale Formation^20^ (Suppl. Fig. S1). The Alum Shale, Miaolingian to early Tremadocian in age, consists of kerogenous black shales (TOC up to 25%) punctuated by centimetre- to decimetre-thick limestone and conglomerate interbeds and calcareous concretions ^20^. The latter, named anthraconites, orsten and stinkstones in the regional bibliography, show different origins. Some of them contain barite cores encrusted or wholly replaced by pyrite, and have been interpreted as pyritic pseudomorphs replacing hydrothermal or diagenetic barite^21^. Other barite-free calcareous concretions display the morphologic, petrographic, mineralogic and geochemical features characteristic of glendonites, and occur embedded in basinal kerogenous shales^22,23^.

On Öland, the Alum Shale Formation is up to 24 m thick at the southern tip of the island, but thins out considerably northward and disappears completely at the northern edge of the island^24,25^. The formation contains several significant episodes of condensation and non-deposition marked by limestone and conglomerate interbeds, such as the Exporrecta Conglomerate Bed (Guzhangian, lower *Lejopyge laevigata* Zone) and the overlying Kakeled Limestone Bed (uppermost Guzhangian‒lowermost Paibian^20,23^) (Suppl. Fig. S1). Samples of carbonate crusts and stellate concretions were recovered from two localities of Öland, Bruddesta and Degerhamn. In the coastal outcrop at Bruddesta (coordinates: N56º59′18′′, E16º46′48′′), the Alum Shale hardly exceeds 2 m in thickness and exhibits significant Furongian gaps. From bottom to top, the Alum Shale Formation can be subdivided into: (i) the Exporrecta Conglomerate Bed, up to 0.4 m thick; (ii) a black shale layer, 0.05‒0.15 m thick; and (iii) the Kakeled Limestone Bed, up to 0.5 m thick^20,23^. The latter bed is a composite limestone, interrupted by intra-bed scouring surfaces, with a trilobite content indicative of the uppermost Guzhangian *Agnostus pisiformis* Zone, whereas laterally, on southern Öland, the Kakeled Limestone contains trilobites of the *Glyptagnostus reticulatus* Zone (Paibian, Furongian)^24–26^. In the second locality, a road section situated in vicinity of Degerhamn town (coordinates: 56°20′43″ N, 16°24′46″ E), the anthraconite concretions and crusts were sampled from the lowermost 0.10‒0.15 m of black shales (Unit 21^26^: Fig. 3), containing trilobites characteristic of the *Parabolina lobata* Zone (Cambrian Stage 10, Furongian), which, according to conodonts^27^ from the same road section, represents the *Proconodontus muelleri* Zone. The Bruddesta section can be taken as an example of a highly condensed and stratigraphically incomplete Guzhangian to Furongian Alum Shale succession occupying a proximal position within the area of black shale accumulation, while the Degerhamn section represents a transition toward stratigraphically complete Alum Shale successions in Scania, southernmost Sweden^24^.

Correlation of the Öland succession with the Miaolingian‒Furongian δ^13^C_org_ curve from the Grönhögen-2015 drill core, some 15 km south of Degerhamn^24^, suggests that the two glendonite-bearing levels described herein are characterized by negative δ^13^C_org_ values_._ These represent the Guzhangian pre-Steptoean Positive Carbon Isotope Excursion or pre-SPICE background (*Agnostus pisiformis* Zone at Bruddesta section), and the negative Top of Cambrian Excursion or TOCE (*Parabolina lobata* Zone at Degerhamn section), respectively. Both the SPICE and TOCE shifts have been interpreted regionally as coinciding with pulses of sea-level fall and increased oxygen level at the seafloor^23,26^.

## Results

### Facies and textures

Three kinds of authigenic carbonates occur in the Exporrecta Conglomerate Bed, the Kakeled Limestone Bed, and the interbedded black shales of the Alum Shale Formation along the western cliffs and coastline of Öland: (1) metalliferous carbonate mounds, (2) glendonitic concretions and (3) lag accumulations.

**Figure 2:**
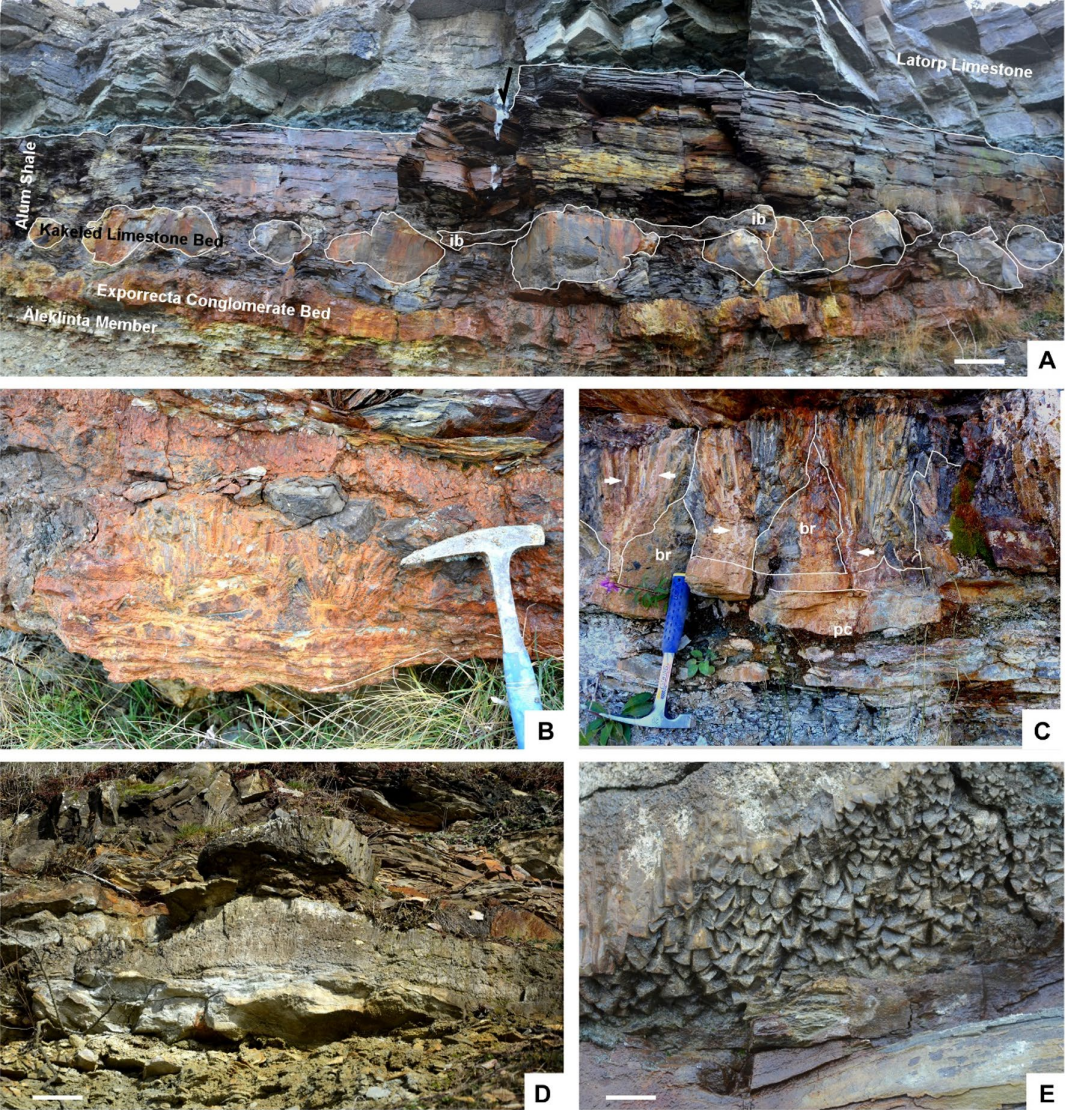
Field aspect of the metalliferous carbonate mounds described in the text. (**A)** General view of Alum Shale/Latorp Limestone faulted contact (arrowed) at Bruddesta highlighting the disconnected aspect of the limestone intervals of the Kakeled Limestone Bed, laterally interrupted by intraformational breccia levels (*ib*); scale bar = 10 cm. (**B)** Close up view of the Exporrecta Conglomerate Bed showing alternations of conglomerate lags encrusted by glendonitic radiating fans. (**C**) View of coalescing stellate glendonite fans forming a blanket, interrupted by whitened hydrothermal veins (arrows); *br* sulphide-bearing breccia lens, *pc* palisade crust. (**D)** Mound of the Kakeled Limestone Bed embedded in the uppermost part of the Alum Shale at Degerhamn; scale bar = 10 cm. (**E)** Basal surface of a glendonitic blanket showing the pyramidal end of the glendonitic crusts at Bruddesta; scale bar = 1 cm.

**Figure 3:**
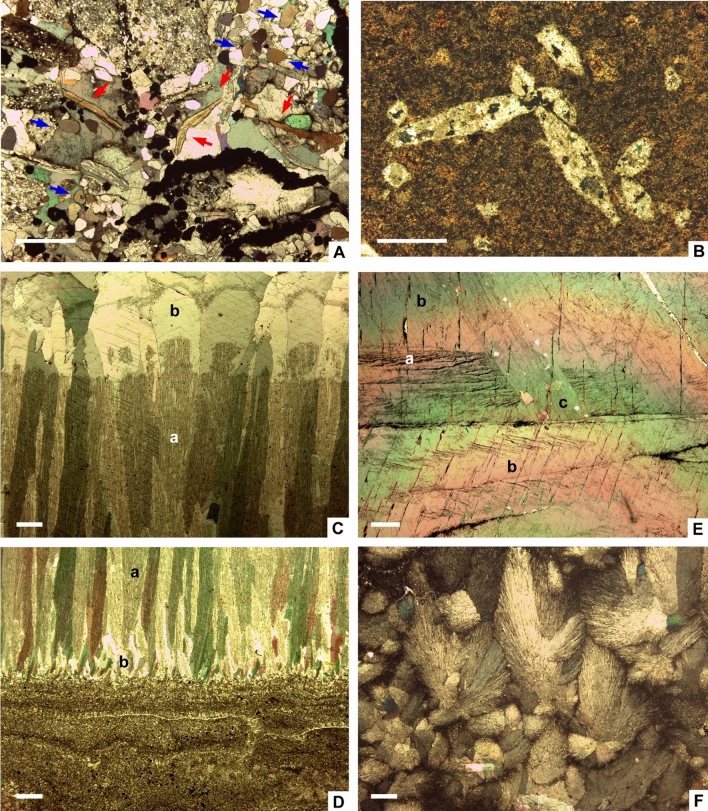
Thin-section photomicrographs (under crossed nichols) of in-situ calcite blankets at Bruddesta. (**A)** Polymetallic basal breccia forming the Kakeled Limestone Bed rich in sulphide crusts and clasts (opaque minerals), subrounded quartz grains, calcite and apatite (marked by blue arrows) clasts, and linguliformean brachiopods (red arrows). (**B)** Calcite pseudomorphs after anhydrite embedded in the limestone rich in zoned crusts of polymetallic sulphides. (**C,D)** Arrangement of cloudy (a) and clean (b) calcite crystals in a crust. (**E)** Crosscutting relationships between *ca1* (a), *ca2* (b) and *ca3* (c) glendonitic phases. (**F)** Feathery crystals of calcite forming the base of a crust; all scale bars = 1 mm.

**Figure 4:**
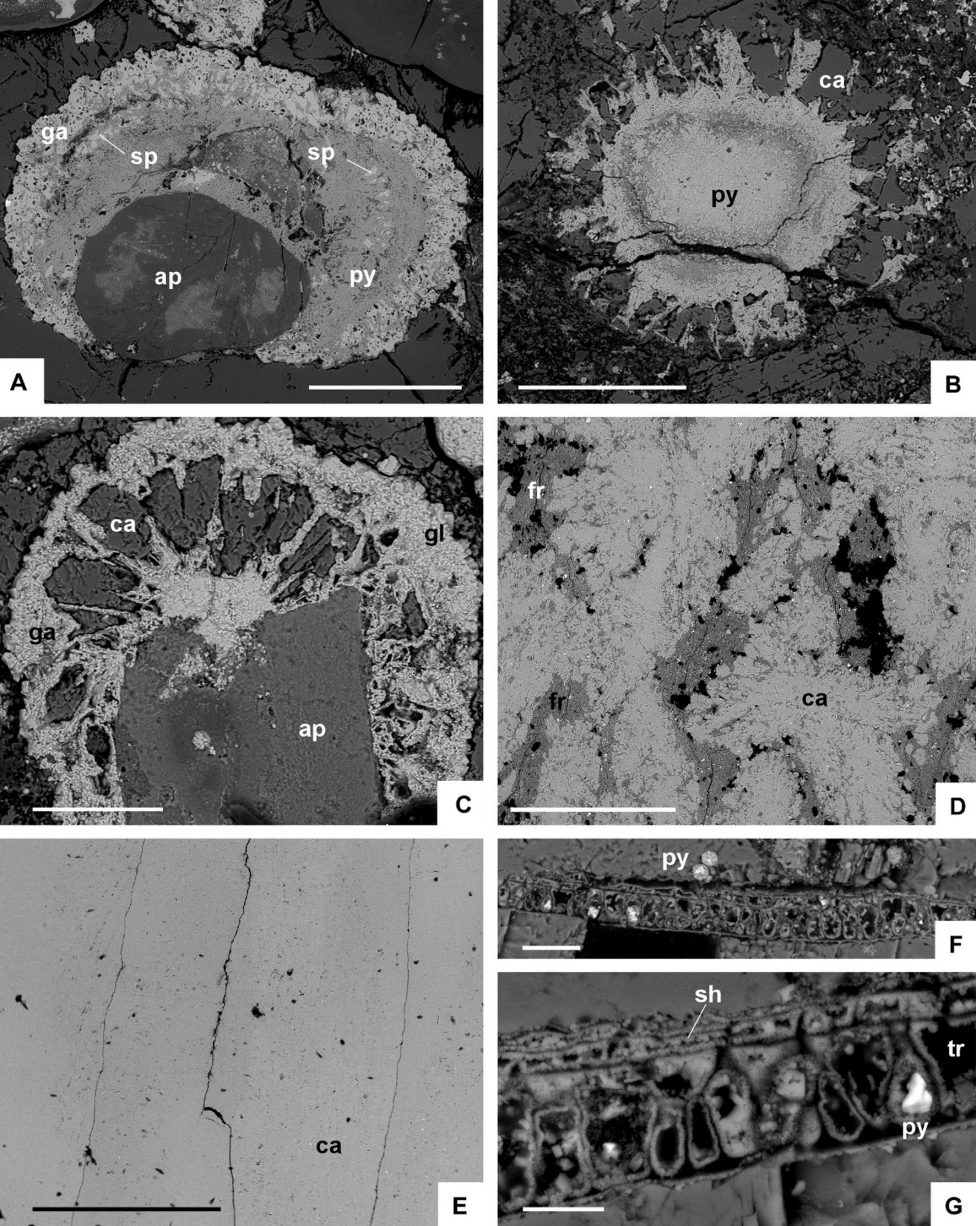
Scanning electron microscopy operating in back-scattered electron (SEM**-**BSE) photomicrographs. (**A)** Polymetallic zoned crustiform band of pyrite including sphaleritic inclusions and growing over a clast of apatite; scale bar = 200 µm. (**B)** Zoned pyrite encrusted by radiating pyrite/glendonite crusts; scale bar = 200 µm. (**C)** Apatite clast encrusted by pyrite and radiating calcite/pyrite crusts; scale bar = 100 µm. (**D)** Glendonitic feathery *ca1* crystals arranged as stellate clusters (ca) separated by a clayey matrix rich in francolite (fr) crystals and strings; scale bar = 500 µm. (**E)** Calcite pseudomorphs after anhydrite, encased in the phosphatic limestone of the Kakeled Limestone Bed; scale bar = 1 mm. (**F,G)** Filamentous cyanobacteria, probably related to *Oscillatoriopsis longa*, with distinct preservation of trichome and sheath; pyrite occurs as framboids in (**F**) and as subhedral crystals infilling the primary porosity of the trichome in (**G**); scale bars = 20 and 10 µm, respectively. *ap* apatite, *ca* calcite (glendonite), *py* pyrite, *sh* sheath, *sp* sphalerite and *tr* trichome.

(1) The most outstanding character of the Alum Shale strata cropping out along the Öland cliffs is the ubiquitous presence of metalliferous carbonate mounds. They occur as sheet-like to domal crusts, up to 1.4 m thick and 8 m across, covering both clayey and carbonate substrates (Fig. 2A–D). These composite mounds comprise clusters of sub-domes (fans in 2D) that occur stacked together bounded by clayey millimetre-thick seams. Each fan comprises a lower polymictic breccia lens overlain and flanked by rosettes, palisade crusts and macro-columnar (1‒50 cm long) crystal fans laterally coalescing in blankets. The basal breccia is clast-supported and rich in angular gravel to cobble clasts, embedded in a poorly sorted sandy matrix and cemented with microgranular mosaics of calcite. Clasts are dominated by shale, sandstone, impure limestone (Fig. 2B), phosphate and opaque (sulphide) minerals (Figs. 3A, 4A,C). The polymictic lag and a part of the overlying calcite palisade crusts are crosscut by networks of sulphide-rich veins. Veins, up to 2 cm thick, occur as swarms of sharply bounded, steeply dipping to vertical fissures (arrowed in Fig. 2C). Vein density ranges from isolated veins to an anastomosing or irregular stockwork with floating wall-rock fragments that also occur as angular clasts in the basal lag. Veins are crosscut and surrounded by vein sand- to granule-sized clasts. X-ray powder diffraction (XRD) and scanning electron microscopy operating in energy dispersive X-ray analysis (SEM–EDS) of the mineralization allowed identification of pyrite (ranging from cubic, subhedral to framboidal shapes), galena, and subsidiary sphalerite and barite (Figs. 3A, 4A–C). The highest enrichment in sulphides occurs in the breccia lags lining the base of each carbonate fan. Limestone beds bearing zoned sulphide crusts, up to 2 mm thick, comprise pseudomorphs of calcite (drusy mosaics) after anhydrite (Fig. 3B).

**Figure 5:**
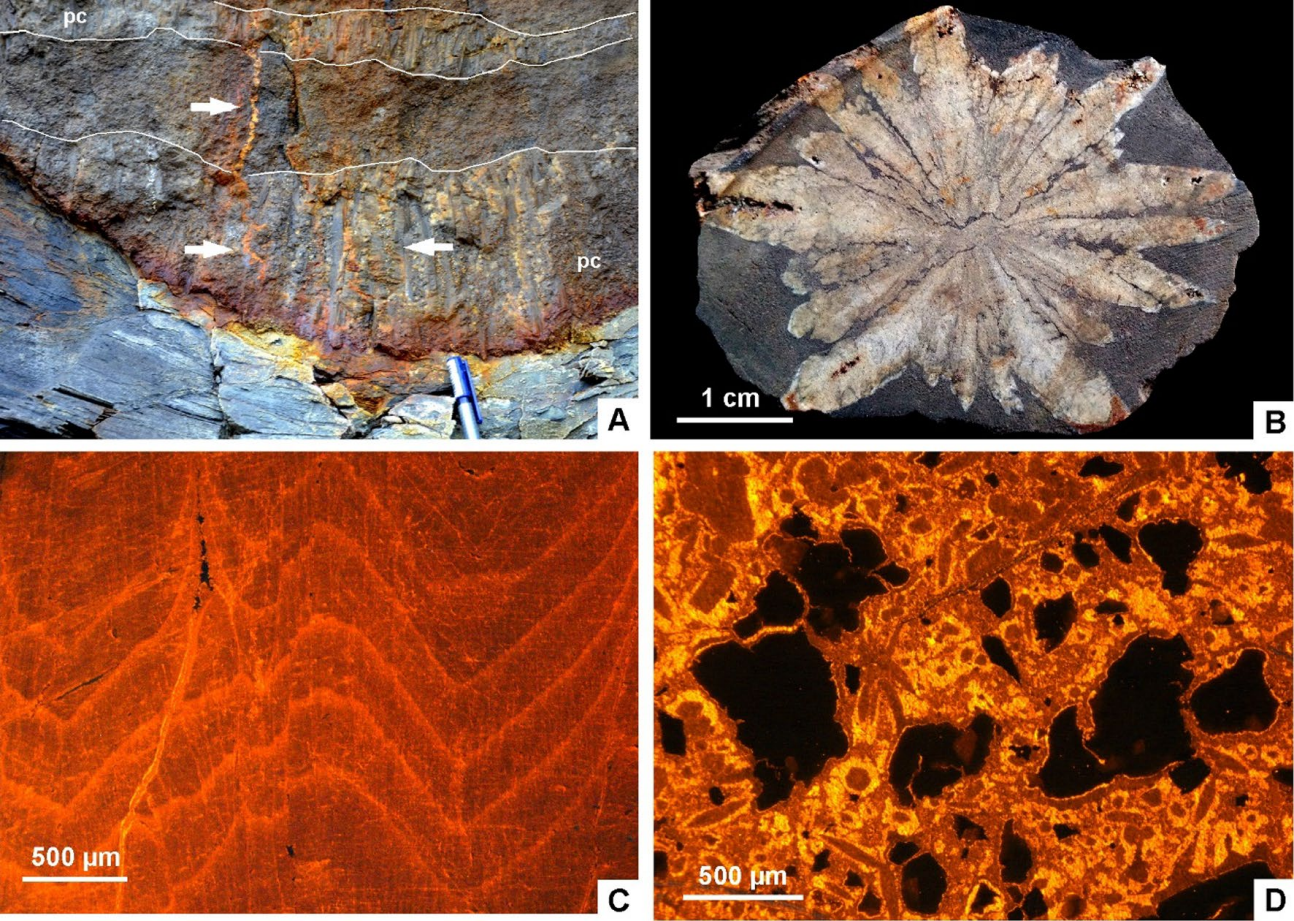
**(A)** Superposition of palisade crusts (pc) in the basal part of the Kakeled Limestone Bed at Bruddesta with arrowed polymetallic veins. (**B)** Slab of a glendonite rosette embedded in the black shale interbeds separating the Exporrecta Conglomerate and Kakeled Limestone beds at Degerhamn. (**C)** Cathodoluminescence thin-section photomicrograph showing the core of a glendonitic aggregate, with zoned dull-orange luminescence, displaying the prismatic chevron-like habit of stellate pseudomorphs. (**D)** CL photomicrograph of a lag accumulation showing a mixture of non-luminescent (polymetallic sulphides) and dull-brownish luminescent glendonitic clasts embedded in a brightly yellowish cement.

Vein networks are sealed by overlying calcite crusts, whose basal part still contains clasts derived from the fissure walls and infills (Fig. 5A). Calcite crusts, up to 50 cm thick, consist of superposed microgranular mosaics grading to fans of feathery crystals (Fig. 3F), and bipyramidal (Fig. 2E) and fascicular, isopachous crusts (Fig. 3C,D), up to 50 cm thick, grading laterally to radiating clusters and botryoids. Palisade crusts and macro-columnar crystals mostly grew perpendicular to substrate. Successive palisade crusts are separated by thin, generally < 1 mm thick, microspar laminae containing scattered detrital silt quartz and clay flakes. Under petrographic microscopy, cathodoluminescence (CL) and SEM, the calcite crusts display at least three generations (Fig. 3E), which include: (i) dirty (silt-rich) microgranular mosaics and radiating blade to feathery, bipyramidal and fascicular crystals (*ca1*) (Fig. 4C–F), which form palisade crusts of impure calcite, encasing silty quartz and polymetallic grains, and clay flakes; individual calcite crystals are lined by clayey and phosphatic (P_2_O_5_ content up to 18 wt% by SEM-BSE) intercrystalline strings and networks (Fig. 4D); (ii) clean crystals (*ca2*) (Figs. 3C,E, 4E), mimicking the textures exhibited by *ca1* but lacking silty impurities, and both infilling intercrystalline porosity and encrusting *ca1* mosaics; and (iii) bladed clean crystals (*ca3*) (Fig. 3E) intersecting and crosscutting the previous phases. The lower palisade crusts are outlined by abrupt acute rhombic to pyramidal terminations. The bands of *ca1* and *ca2* crystals show dull, reddish and zoned luminescence, and are commonly separated and intersected by distinct, moderately luminescent brownish *ca3* crystals. Crystals *ca1* and *ca2* generally display a uniform extinction under cross polarized light, are non-ferroan, and cloudy to transparent under transmitted light, respectively. Locally, small (< 10 μm) framboidal pyrite occurs lining the contact between palisade crystals.

(2) Glendonitic concretions occur as single to composite crystal pseudomorphs exclusively encased in black shales, commonly growing as radiating and intersecting crystal blades forming stellate and rosette clusters, about 5‒20 cm across (Fig. 5B). Under petrographic, CL and SEM microscopies (Fig. 5C), the concretions exhibit a centripetal array of internal textures, ranging from (i) macrozoned or chevron-like textures displaying a millimetre-scale zoning of prismatic crystals at the core, to (ii) homogeneous (or unzoned) blades that finally grade peripherally to (iii) microgranular mosaics of calcite. Macrozoned blades are lath-shaped, inclusion-rich calcite crystals, either distinctly zoned or with a clear distinction from rim to core. They show undulatory extinction under cross-polarized light and, under CL, they are dully luminescent with brightly luminescent outlines marking crystal contacts. Unzoned crystals form mosaics of sparry calcite infilling inter-crystalline pores, and are mostly non-luminescent. The granular texture consists of equant to bladed, sand-sized, calcite crystals, which are overgrown by variably sparry and spherulitic calcite cements and, under CL, the crystals are brightly luminescent, with some zonation marked by sharp changes from red to orange cements (Fig. 5C). The margins of glendonite crystals terminate sharply against the enclosing sediment. Staining with Alizarin Red-S and potassium ferricyanide shows that the zoned crystals and many of the unzoned and granular overgrowths are non-ferroan.

(3) Lag accumulations are thin (< 10 cm) but laterally extensive layers that rest on scouring surfaces that generally truncate the underlying homogeneous black shales. Clasts are poorly sorted and their size ranges from less than 1 mm up to 4 cm. They display crude upward-fining textural trends. Clasts consist of subangular calcite and glendonite-derived unzoned (feather-shaped) and microgranular calcite fragments, with brightly luminescent orange to yellowish luminescence (Fig. 5D), and subsidiary sulphide clasts derived from the above-reported carbonate counterparts. Primary interparticle porosity is commonly occluded with microsparite to sparite, in some cases forming equant, blocky mosaics.

### Raman analysis of carbonate crusts

Laser-spot Raman analysis of the carbonate crusts allowed identification of two CaCO_3_ polymorphs: calcite and subsidiary vaterite. The Raman spectra show the typical CO_3_^2−^ vibration modes *v*_1_ (1085 cm^−1^), *v*_3_ (1435 cm^−1^), *v*_4_ (711 cm^−1^) and translational lattice modes (T = 284 cm^−1^) of calcite. The subsidiary presence of vaterite is suggested by the vibration mode *v*_1_ (1086 cm^−1^), *v*_3_ (1465 cm^−1^) and *v*_4_ (664 cm^−1^). A minor peak at 353 cm^−1^ is assigned to Ca-O lattice vibrations. The spectra were compared to in-house reference material^28,29^. Peaks at ~ 2900‒3700 cm^−1^ are referred to the OH stretching transition of water^30^ (Repository Data).

### Carbon and oxygen isotope composition

**Figure 6:**
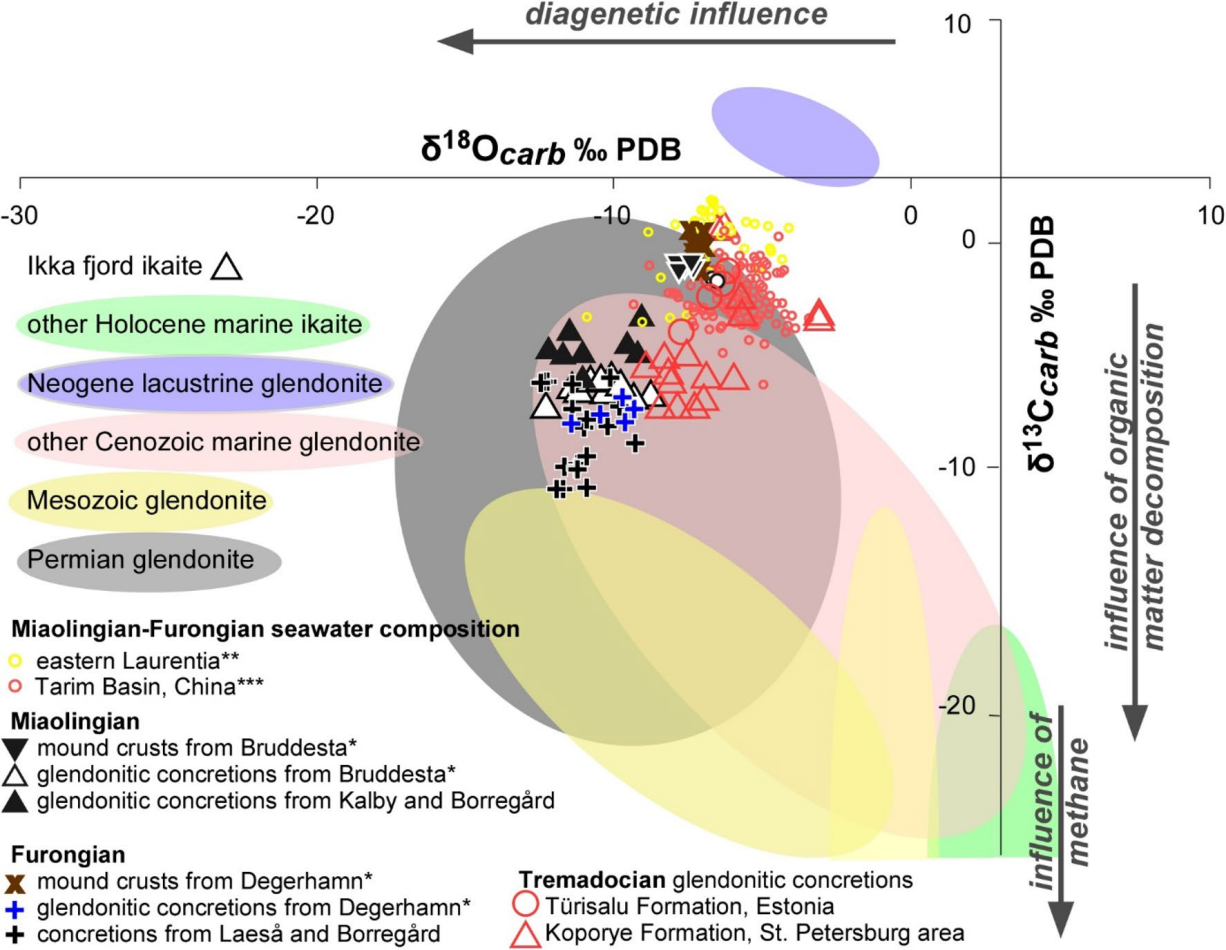
Stable isotope dataset plot for mound crusts and glendonitic concretions from the Miaolingian (including concretions from Kalby and Borregård^22^), Furongian (adding concretions from Laeså and Borregård^22^) and Tremadocian^31,32^ of the Baltoscandian Basin; shaded areas^33–36^; asterisk: new data from Repository Data; double asterisk: comparative data from Laurentia, and triple asterisk: from China^37,38^.

The *ca1* and *ca2* phases of the glendonitic concretions display broad ranges of δ^13^C_carb_ (ranging from − 4.7 to − 8.5‰) and narrower ranges of δ^18^O_carb_ (from − 9.1 to − 12.4‰) values^22,this work^ (Fig. 6; Suppl. Table 1). In contrast, the mound crusts show narrow ranges of δ^13^C_carb_ (from − 1.1 to + 1.6‰) and δ^18^O_carb_ (from − 6.5 to − 7.6‰) values, irrespective of the *ca1–ca2* phase analysed (Fig. 8).

### Microthermometric analysis of fluid inclusions in carbonate crusts

Fluid inclusions (FI) were investigated in mound crusts from the Kakeled Limestone Bed at Bruddesta (Suppl. Table 2). Cloudy calcite crystals (*ca1*) and mosaics of clean calcite crystals (*ca2*) are rich in primary fluid inclusion assemblages or FIAs (i.e., scattered inclusions, small clusters and trails following growth zones) that display irregular outlines and variable sizes (5‒44 µm). At room temperature, they are mostly all-liquid but the largest inclusions show two-phase (liquid + vapour) FI with liquid percentages about 95%. The all-liquid FI nucleate a bubble inside with cooling. All FI freeze at temperatures ranging from ‒52 to ‒74 °C, usually around ‒60 °C (*T*_n_). Upon reheating, the first melting temperature (*T*_fm_) is observed at ‒67 to ‒50 °C, and the final melting of ice (*T*_mice_) at ‒23.7 to ‒11.2 °C. FI homogenization (*T*_h_) occurs to the liquid usually from 59 to 85 °C. The low *T*_fm_ of the measured FIAs indicates the presence of bivalent cations being close to the eutectic temperature of the H_2_O–NaCl–CaCl_2_ system^39^. The fact that both *ca1* and *ca2* share FIAs with similar *T*_fm_, *T*_mice_ and *T*_h_ suggests that *ca1* recrystallized under the same fluid that precipitated *ca2*, probably infilling the primary porosity yielded by the ikaite-*ca1* transformation. In the absence of pressure estimates, these *T*_h_ can be interpreted as a minimum entrapment temperature^40^, providing a minimum temperature for *ca2* precipitation and *ca1* recrystallization.

Fissures occluded with *ca3* mosaics contain another type of FIAs. They are primary to the fracture filling, as manifested by their long axis aligned to the direction of the fracture. Their size ranges from 20 to 70 µm, and are all-liquid or all-gas or very rich in gas. In the FI with enough liquid, the freezing (*T*_fm_) occurs between ‒44 and ‒40 °C, the first melting around ‒21 °C and the *T*_mice_ between 2.3 and ‒1.8 °C. *T*_fm_ ‒21 ºC is indicative of a H_2_O–NaCl system^40^, and salinities being 3.06‒3.87 NaCl wt% equivalent^39^.

### Sulphur isotope signatures of polymetallic sulphides

As stated above, the basal lags of the metalliferous carbonate mounds display accumulations of hydrothermal sulphides sourced from the veins affecting the lower calcite crusts and sealed (so post-dated) by the upper calcite crusts. Pyrite occurs as single euhedral grains and framboids (up to 20‒100 µm in diameter), in some cases forming centimetre-sized pockets. Galena occurs as subhedral to anhedral grains with interstitial pyrite and sphalerite inclusions.

**Figure 7:**
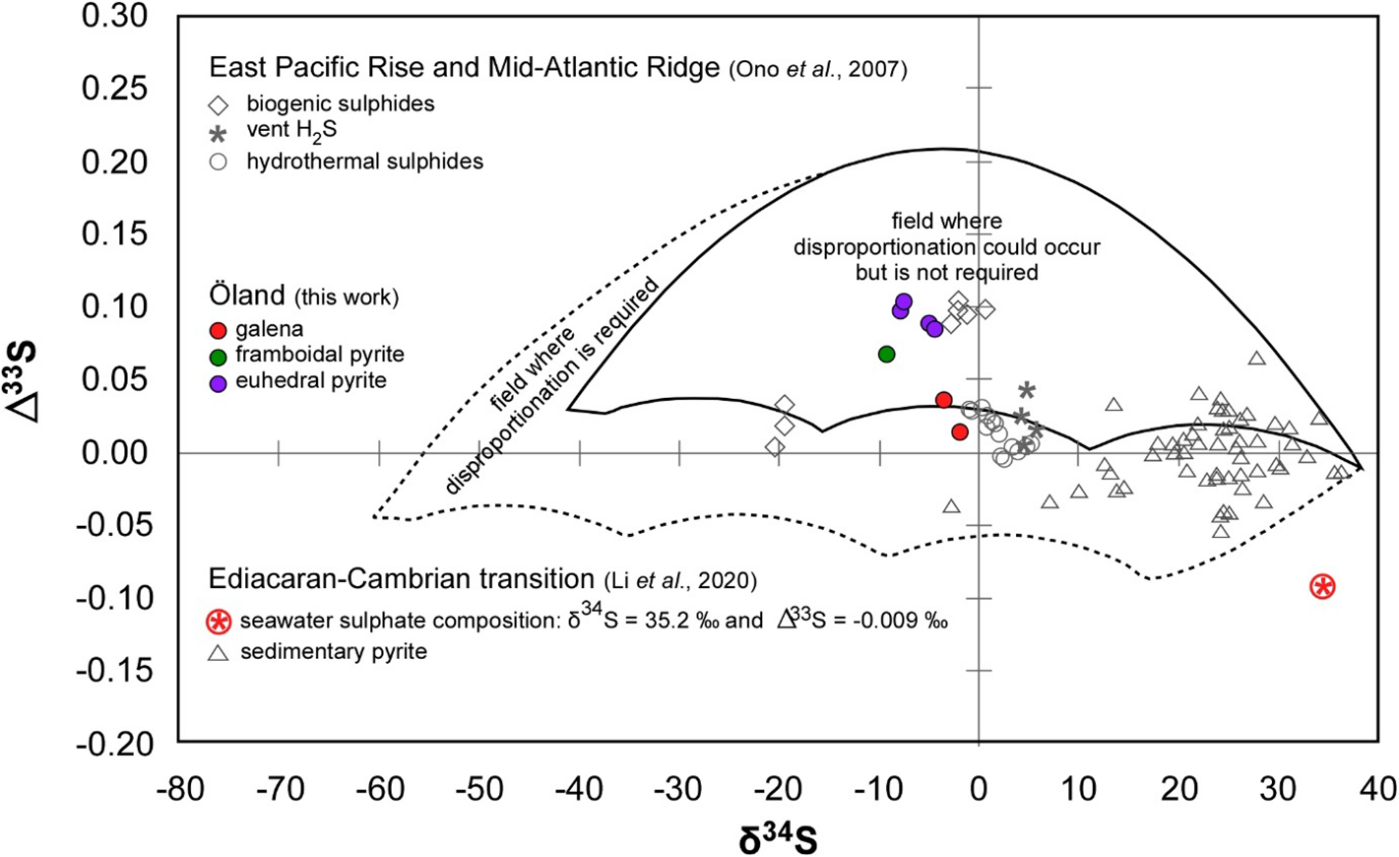
Plot of δ^34^S vs. ∆^33^S for the Bruddesta pyrite and galena precipitates, compared to present-day biogenic and hydrothermal sulphide and vent H_2_S from the East Pacific Rise and Mid-Atlantic Ridge^41^ and sedimentary pyrites from the Ediacaran‒Cambrian transition^42^. The model fields illustrate the field of S-isotope compositions produced by sulphate reduction where disproportionation could occur but not required (black outlined field) and sulphate reduction combined with sulphur disproportionation (black dotted line field)^43,44^.

Analyses of the four stable sulphur isotopes (^32^S, ^33^S, ^34^S and ^36^S; Suppl. Table 3) from the polymetallic sulphides encased in the *A. pisiformis* Zone of the Kakeled Limestone Bed at Bruddesta yielded δ^34^S values ranging from ‒2.08 to ‒9.33 ‰, with ∆^33^S [= (^33^S/^32^S)_sample_/(^33^S/^32^S)_reference_ − ((^34^S/^32^S)_sample_/(^34^S/^32^S)_reference_)^0.515^] ranging from 0.014 to 0.103 (Fig. 7). Among different sulphur species, two galena samples display the most ^34^S-enriched values (δ^34^S: ‒2.08 ‰ and ‒3.56 ‰) and minimal ∆^33^S values (0.014 and 0.036), respectively. The framboidal pyrites exhibits the most ^34^S-depleted value (δ^34^S: ‒9.33‰) with ∆^33^S of 0.068 (Fig. 7; Suppl. Table 3).

## Discussion

### Formation of metalliferous precipitates

The metalliferous carbonate mounds consist of a lower polymictic breccia-to-conglomerate base, crosscut by hydrothermal veins, capped by coalescent crystal fans (in 2D). Clasts composed of shale, sandstone and limestone reflect the typical composition of the basal black shales and the laterally equivalent Exporrecta Conglomerate and Kakeled Limestone beds (Fig. 2B).

The δ^34^S values from the Bruddesta pyrites (from ‒4.61 to ‒9.33‰) are more negative than those of the pyrites embedded in contemporaneous strata from the *A. pisiformis* Zone of the Alum black shales (‒0.4 to + 10.2‰^45^), which have not been influenced by synsedimentary hydrothermal processes. The relatively positive δ^34^S values for the latter pyrites were argued to have resulted from very low seawater sulphate concentration, as a result of Rayleight fractionation under diffusion limited conditions within the sediment^43^. Similar values could be attained under high seawater sulphate concentrations due to limited availability of reactive iron and organic matter^46^, but both components were widely available in the Baltoscandian Basin. The negative δ^34^S values with positive ∆^33^S values for the pyrites of the Öland exhalates, plotting in the sulphate reduction field (SRF)^44,45^, probably reflect mixtures of biogenic sulphur and deeply sourced sulphur (Fig. 7). In fact, thermal sulphate reduction (an equilibrium process that depends primarily on temperature and pressure) and microbial sulphate reduction (a kinetic process that mainly depends on reaction rates) are controlled by two different mechanisms. During high temperature water–rock interactions, sulphate-bearing hydrothermal fluids circulated through surrounding rocks that resulted in thermogenic reduction of sulphate to ^34^S-depleted sulphide, a temperature-dependent reaction^47,48^. It is also likely that microbial sulphate reduction of seawater sulphate would have produced ^34^S-depleted sulphide. Though the limited data do not allow us to quantitatively evaluate each fraction of biogenic and hydrothermal pyrite, the most positive δ^34^S with lowest ∆^33^S values for galena suggest a predominant sulphur source derived from the inorganic reduction of hydrothermal sulphate^39^. In contrast, framboidal pyrite with the most negative δ^34^S with positive ∆^33^S values suggest that sulphur source for framboidal pyrite formation may have been dominantly from microbial sulphate reduction^45^.

### Formation of carbonate mounds

After the end of hydrothermal activity and deposition of its sulphide clast products, the framework of the metalliferous carbonate mounds was sealed (so post-dated) by rosettes, palisade crusts and macro-columnar crystal fans of carbonate, laterally coalescing into blankets. The acute rhombic to pyramidal terminations of the recrystallized calcite, and the phosphatic composition of their matrix envelopes point to original ikaite crystals^5^, whereas the overlying clean crystals of calcite displaying fibrous to columnar shapes, and lacking significant contents of phosphate, may reflect original precipitation of aragonite.

As in the case of the metalliferous carbonate mounds, the texture of the glendonitic concretions also represent at least three calcite generations, where the macrozoned texture (Fig. 5C) is interpreted as preservation of the original ikaite-derived texture^49^, and the unzoned and granular ones as the final generation of diagenetic calcite growth^32^. Their CL patterns of the three calcite generations reflect a progressive increase and zonation in luminescence: the increase in luminescence activators may be related to changes from relatively oxidizing (or suboxic) to more reducing pore waters. The ikaite-glendonite replacement generated porous pseudomorphs, subsequently occluded with cement phases that overgrow the replacement phase to form the unzoned and granular textures. The observed relationships between the different calcite phases suggest multiple stages of dissolution–recrystallization and replacement (either complete or partial) of pre-existing types of calcite. Finally, lag accumulations reflect the influence of strong bottom currents, mainly related to storm processes, reworking the aforementioned authigenic palisade crusts and stellate clusters.

The broad range of δ^13^C_carb_ (ranging from − 4.7 to − 8.5‰) and δ^18^O_carb_ values (from − 9.1 to − 12.4‰) of the *ca1* and *ca2* phases in the glendonite concretions reflects variation in carbon sources, at least two generations of calcite pseudomorphosing after ikaite. The δ^13^C_carb_ values of the glendonite concretions, lower than those of the mound crusts, reflect carbon sources derived from microbial degradation of organic matter. The oxygen isotope signal in the pseudomorphosed glendonite crystals is more likely influenced by exchange reactions, especially during recrystallization and burial diagenesis at higher temperatures.

Tremadocian glendonitic concretions from the Türisalu and Koporye formations of Estonia and the surroundings of St. Petersburg, Baltica^31,32^ show different ranges of δ^13^C_carb_ (from − 7.2 to + 0.6‰), reflecting more negative δ^13^C_carb_ values than those yielded by the Cambrian glendonites reported here (Fig. 6). Late Tremadocian to Floian glendonite-free limestone interbeds from the Latorp Formation (Jämtland, Sweden), a lateral equivalent of the overlying glendonite-free glauconitic sands of the Leetse Formation, have yielded δ^13^C values more positive than those of the study glendonites (ranging from + 0.27 to − 0.73‰)^50^, reflecting isotope values closer to seawater composition.

In contrast, the narrower range of δ^13^C_carb_ and δ^18^O_carb_ values in the mound crusts reflects a homogeneous carbon source and diagenetic preservation. The δ^13^C_carb_ data suggest that the hydrothermal fluids related to this precipitation entrained dissolved inorganic carbon (DIC) whose isotope signature was higher than that of the contemporaneous glendonite concretions by ca. 6.3‒8.5‰. This offset probably represents a mixture of the DIC released during hydrothermal emission, and thermal degradation of organic matter from underlying kerogenous shales. The offset in δ^18^O, between − 7.6 and − 6.5‰, is less pronounced than that of δ^13^C, which contrasts with the supposed opposing differences in palaeotemperatures of precipitation, as documented below, and would reflect isotope re-equilibration after recrystallization at higher temperatures.

Despite the above-reported influence of microbial activity and biogenic degradation of organic matter, the carbon isotope values reported in Bruddesta and Degerhamn broadly follow the values related to the contemporaneous positive trend prior to the onset of the SPICE and the negative TOCE (or HERB) chemostratigraphic shifts from subtropical areas, respectively. The magnitude of the SPICE excursion is generally consistent (an ~ 4‰ increase), though the peak δ^13^C_carb_ values are quite variable (ranging from + 0.4 to + 5.9‰), throughout sections located between 30 and 60°S paleolatitudes record δ^13^C_carb_ values ~ 1 to 2‰ lower than those from lower paleolatitudes^51^. The TOCE shift is characterized by a negative peak of δ^13^C_carb_ values at − 3‰ to − 4‰^40^. The TOCE peak is well calibrated in Öland^24^, but needs to be confirmed worldwide.

### Evolution of hydrothermal activity in a tectonosedimentary context

During Miaolingian‒Furongian times, synsedimentary extensional fault pulses in the semi-enclosed Baltoscandian Basin favoured the episodic development of basement reworking (Exporrecta Conglomerate Bed) and shelly carbonate production on palaeo-highs (Kakeled Limestone Bed), surrounded by deposition of kerogenous black shales. Erosion and non-deposition in horst-related settings controlled the record of significant gaps. The basin developed water column stratification, where plumes of buoyant fluid were trapped when their buoyancy with respect to the environment reversed, fluids became heavier than their surroundings and gravitational forces brought them to a halt, favouring the lateral dispersion of effluents and sulphide particle settling^13,16^. As a result, plumes of micro-particulate metal oxides favoured precipitation out of hydrothermal fluid venting into an oxic water column. Under stratification of the water column, fallout took place only when the reduced metal ions get oxidized at the redox cline. The settling particles then formed a metalliferous sediment that mixed with a kerogenous-dominant seafloor^53^ episodically interrupted by brief oxygenation events^54^. Considering the water column stratification, the reservoirs of dissolved inorganic carbon (DIC) and organic carbon (DOC) were mainly controlled by bacterial degradation of benthic organic matter, which supplied the DIC required for the episodic massive precipitation of ikaite concretions, subsequently pseudomorphosized to glendonite.

**Figure 8:**
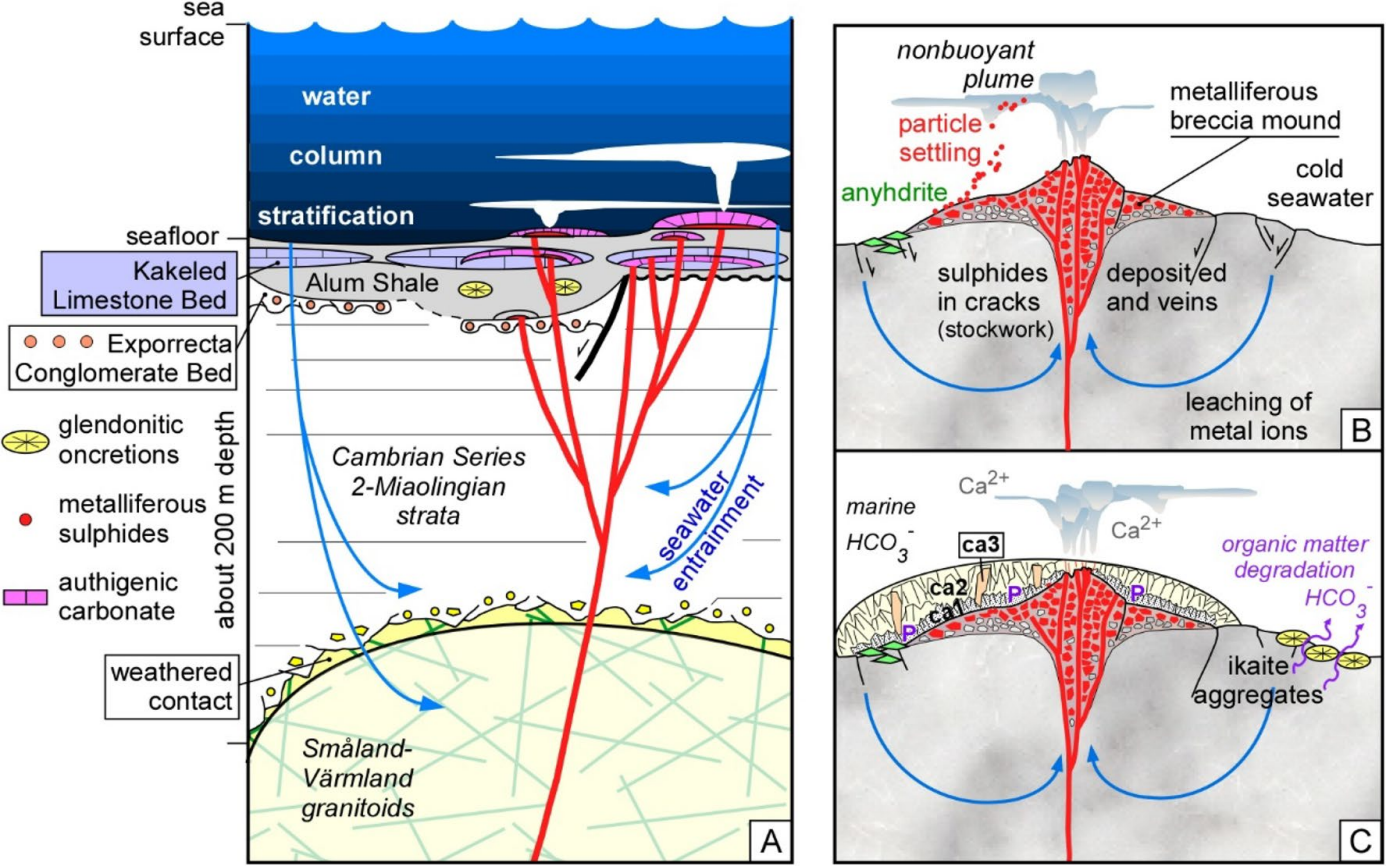
**(A)** Schematic drawing of the Öland hydrothermal system encased in the Alum Shale Formation showing the different components and processes described in the text. (**B)** First phase characterized by deposition of pyrite, galena and chalcopyrite as vein stockworks, reworked angular clasts and particle settling from a nonbuoyant plume, triggered by vigorous venting of slightly acidic hydrothermal fluids with temperature > 100 ºC. (**C)** Second phase of a metalliferous carbonate mound, influenced by slightly alkaline hydrothermal fluids below 100 ºC, and showing overlying crusts of turbid ikaite (now *ca1*) rich in phosphate (*P*), limpid aragonite (now *ca2*), and recrystallized crystals of calcite that also occur occluding fissures (*ca3*). The hydrothermal system is separated from hydrothermal-free substrates of kerogenous shales encasing ikaite (now glendonitic) concretions recording distinct carbon isotope values controlled by thermal degradation of the organic matter.

The metalliferous carbonate mounds of Öland may reflect the onset of hydrothermal processes in a heavily sedimented rift to post-rift framework^55^, where the long-term heat retention afforded by a thick sediment cover (about 200 m thick in our case study), as well as the entrapment and insulation of vent fluids, may account for the episodic development of sediment-hosted lead–zinc deposits^56^. Leaching of footwall rocks by hydrothermal fluids interacting with the granitic and the Cambrian Series 2-Miaolingian siliciclastic strata basement would be triggered by tectonic events. In the neighbouring island of Äspö, a distinct hydrothermal alteration of the Småland-Värmland granitoid basement is related to saussuritization, albitization and replacement of plagioclase by K-feldspar processes made under acidic conditions, resulting in the formation of secondary iron oxides and calcium-bearing minerals, such as calcite, epidote, pumpellyite and prehnite^57^. Both the granitic and terrigenous strata that form the basement of the Alum Shale Formation are candidates for the provenance of the Pb‒Zn‒Fe stockwork and the authigenic carbonate that form the metalliferous carbonate mounds (Fig. 8). At seafloor pressures, anhydrite dissolves back into seawater when temperatures drop below about 150 ºC^58^, which would offer a palaeotemperature proxy for the precipitation of the sulphate, now recognizable by its pseudomorphs (Fig. 3B).

Metal sulphides precipitated and formed dense black clouds of particles that are encased in the breccia lags encrusted by the first step of *ca1-*bearing palisade crusts. Gradations of these first emissions toward fluids with lower temperatures and neutral to lightly alkaline conditions, would have favoured precipitation of ikaite (Table 1). The δ^13^C range of the authigenic carbonates is similar to contemporaneous shelly limestones^23^ (Fig. 6). Its offset from the δ^13^C values in contemporaneous glendonitic concretions is related to the influence, in the latter, of the thermal degradation of organic matter encased in the underlying kerogenous shales. Mixing of warm, calcium-rich vent fluids with cool, bicarbonate-rich seawater would have resulted in the precipitation of ikaite-aragonite.

**Table 1:**
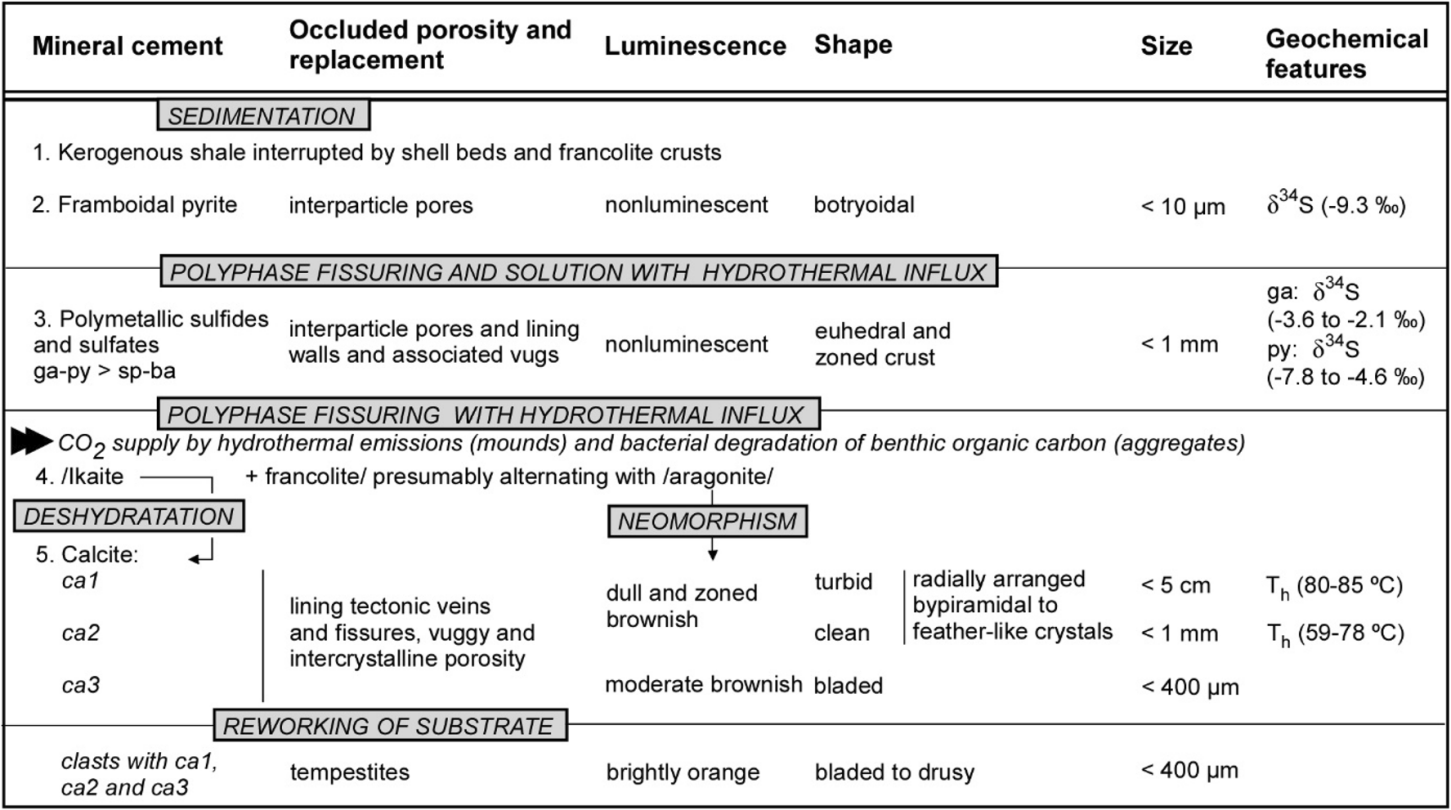
Idealized synsedimentary and early-diagenetic succession affecting the carbonate crusts from Öland.

*ba* barite, *ca* calcite, *ga* galena, *py* pyrite, *sp* sphalerite.

Three chemical factors controlled the precipitation of ikaite vs. aragonite: (i) under high supersaturation conditions and in the presence of magnesium and phosphate (seawater), which inhibited the precipitation of aragonite-calcite, preferentially feathery crystals and crystal fans of ikaite formed, whereas idiomorphic calcite crystal grew probably slowly^59^; (ii) kinetic barriers of ikaite vs. calcite/aragonite precipitation were also controlled by temperature^60^; and (iii) the influence of cyanobacterial blooms, which commonly deplete the dissolved CO_2_ concentration and increase water column pH to alkaline levels. These are evidenced by the massive accumulation of phosphatized cyanobacterial filaments in the *Agnostus pisiformis* Zone of the Kakeled Limestone Bed. This unit represents an Orsten Konservat-Lagerstätte with a preservation controlled by the incrustation and impregnation of a thin external layer of apatite (francolite) encrusting microbial filaments. After acid etching, this level has yielded a broad variety of arthropod^61^ and cyanobacterial microfossils, such as annulated cylindrical filaments (Fig. 4F) referred to *Oscillatoriopsis longa*^62^. The three-dimensionally preserved specimens comprise hollow, open-ended threads with traces of annulation on the surfaces. In contrast, the phosphatized filaments illustrated from the polymetallic ore breccias show a distinct preservation of the inner trichome (row of cells) and enveloping sheaths (Fig. 4F–G).

The first phases of ikaite precipitation are represented by mosaics of impure, microgranular, feathery (mimicking delicate, needle-like aragonite fans following the direction of fluid flows)^8^, and *ca1* crystals, encasing and lined by phosphate clasts and microcrusts, respectively (Fig. 5D). Provided alkalinity was sufficiently high over the range in pH to maintain supersaturation with respect to calcite and aragonite, ikaite precipitation would have been dominant during the first steps of crust growth under significant concentrations of dissolved phosphate^57^. In addition, ikaite precipitation in kerogenous sediments and its subsequent transformation to glendonite has been reported to be closely associated with the decomposition of organic matter (releasing bicarbonate during anaerobic oxidation), mostly controlled by microbial sulphate reduction. Thereby it has to be taken into account that organoclastic sulphate reduction, which leads to a lower pH relative to seawater, also may lower supersaturation with respect to carbonate minerals^63,64^. Sulphate reduction is evidenced by the precipitation of framboidal pyrite in the pore space between some replacive low-Mg calcite crystals, feathery to bipyramidal in shape, which point to original ikaite precipitation. *T*_h_ of fluid inclusions encased in palisade crusts (ranging from 65 to 78 ºC) provide minimum entrapment temperatures for *ca2* precipitation and *ca1* recrystallization. These palaeotemperatures would favour precipitation of a mixture of ikaite and aragonite/calcite (directly controlled by phosphate concentration), as represented by a transition zone in the phase diagrams for calcium carbonate nucleation temperatures as a function of pH^60^. Finally, the precipitation of fans of clean fascicular, isopachous crusts (*ca2*), not associated at the micrometric scale with phosphatic co-precipitation, may represent precipitation of aragonite and low-Mg calcite. The subsequent recrystallization of ikaite/aragonite overgrowths to low-Mg calcite complicates the correct distinction between all the original crystalline precipitates (Fig. 8).

## Conclusions

The hydrothermal polyphase origin and relationship with tectonic events of Cambrian metalliferous carbonate mounds cropping out on the island of Öland, Sweden, are reported. The mounds represent two massive precipitation events characterized by polymetallic ore deposits encrusted by mixtures of ikaite and aragonite, now recrystallized to glendonite/calcite and subsidiary vaterite. The hydrothermal activities reflect variations from highly reduced, acidic, metalliferous springs at ~ 150 ºC to slightly alkaline, calcium-rich, warm (< 100 ºC) fluids controlling the precipitation of authigenic carbonate. Water column stratification of the Baltoscandian Basin would favour trapping of non-buoyant hydrothermal plumes. The hydrothermal veins mostly formed from fluids originated from deeply convecting fluids, controlled by syntectonic extensional pulses. The composite mounds, up to 1.4 m thick and 8 m across, comprise clusters of sub-domes that appear stacked together. Each fan consists of a lower polymictic breccia lag overlain and flanked by rosettes, palisade crusts and columnar crystal fans that laterally coalesce in blankets. Basal ore concentrations include pyrite, galena, and subsidiary sphalerite and barite, sourced from vertical vein fissures. Stable sulphur isotopes from the polymetallic sulphides reflect mixtures of biogenically induced and hydrothermal pyrite. During high temperature water–rock interactions, sulphate-bearing hydrothermal fluids circulated through surrounding rocks and inorganic reduction of sulphate to ^34^S-depleted sulphide occurred, followed by an equilibrium isotope fractionation that was temperature-dependent.

The mound crusts show narrow ranges of δ^13^C_carb_ and δ^18^O_carb_ values, which contrast with those from laterally equivalent glendonite concretions, encased in black shales. The concretions display broader ranges of δ^13^C_carb_ and narrower ranges of δ^18^O_carb_. This offset suggests a mixture of the alkalinities released during hydrothermal venting and thermal degradation of organic matter, respectively. *T*_h_ of fluid inclusions, ranging from 65 to 78 ºC, provide minimum entrapment temperatures for carbonate precipitation and early recrystallization.

## Samples and methods

### Microscopy

Samples were petrographically characterized using a combination of methods, including transmitted light microscopy with thin-sections stained by Alizarin Red S and Potassium Ferricyanide, scanning electron microscopy (SEM) operating in back-scattered electron (BSE) image and energy dispersive X-ray (EDS) analysis, and separate cold cathodoluminescence microscopy. Scanning electron microscopy analysis was made at Museo Nacional de Ciencias Naturales and Cathodoluminescence (CL) at Complutense University, Madrid. Analytical results of back-scattered electron imaging and EDS analyses display an error of ± 5 to 7%. The qualitative mineralogical composition of some complex samples was determined by the X-ray powder diffraction method (XRD). Interpretation of cementation history was made by distinguishing cement types based on colour, brightness, luminescence patterns, cement morphology and cross-cut relationships. Complex zonation of cements (revealed by CL, BSE and EDS) allowed correlation of cement zones between samples.

### Fluid inclusion microthermometry

Fluid inclusion microthermometry was used to characterize fluid composition and entrapment temperatures. The petrographic studies of fluid inclusions on doubly polished thin sections were performed using a Linkam THMSG.600 (Linkam Scientific, Tadworth, UK) fitted on a binocular Olympus BX51 microscope (Olympus, Tokyo, Japan) at the Complutense University of Madrid. Identification of fluid inclusion assemblage (FIA) followed Goldstein and Reynolds’ method^37^. The stage was calibrated with synthetic fluid inclusions, including triple point of CO_2_, melting point of H_2_O and critical point of H_2_O.

### Raman spectroscopy

The non-intrusive and non-destructive Raman spectroscopy technique (confocal Raman microscopy, Thermo Fisher DXR spectrograph, Waltham, MA, USA) of the Museo Nacional de Ciencias Naturales in Madrid was used to characterize the composition of the inclusion-bearing carbonate crusts^27^. The light at 532 nm of a frequency doubled Nd: YVO4 DPSS solid laser (maximum power 30 mW) was used for excitation. Spectral data were analyzed with Thermo Scientific OMNIC Series Software.

### Carbon and oxygen isotope analysis

Carbon and oxygen isotope analyses were carried out on carbonates, which were removed by dental drill under a binocular microscope and analysed at the Erlangen University. Carbonate powders were reacted with 100% phosphoric acid at 75 °C using a Kiel III carbonate preparation line connected to a Thermo-Finnigan 252 mass spectrometer (Thermo Fisher Scientific). All values are reported in ‰-relative to Vienna Pee Dee Belemnite (VPDB) by assigning a δ^13^C value of + 1.95‰ and a δ^18^O value of ‒2.20‰ to NBS19; reproducibility was checked by replicate analyses of laboratory standards and it was better than ± 0.01 to 0.02‰.

### Sulphide extraction

Sulphide clasts from carbonate samples were extracted using chromium reduction and converted to silver sulphide. During this procedure, the product H_2_S was carried by nitrogen gas through a condenser and a bubbler filled with milli-Q water, and collected as zinc sulphide by reaction with a slightly acidic Zn-acetate solution. The zinc sulphide was made to react with silver nitrate to yield silver sulphide, which was collected by centrifugation and washed with successive rinses of milli-Q water, ammonium hydroxide solution and milli-Q water.

### Sulphur isotope analysis

Silver sulphide (Ag_2_S) was converted to SF_6_ by a fluorination reaction with a fivefold excess of F_2_ at 250 °C for 8 h in a Ni reaction vessel. After the reaction, product SF6 was condensed from the residual F_2_ into a liquid-nitrogen-cooled trap (− 177 °C). The F_2_ was removed to another part of the manifold where it was passivated by reaction with hot KBr. The SF_6_ was subsequently thawed to room temperature and then cooled to − 111 °C to condense contaminants, such as trace HF, before it was transferred to the injection loop of a gas chromatograph, which was cooled to − 177 °C. Gas chromatograph purification of SF_6_ was undertaken, at the University of Science and Technology in Hefei, using a composite column consisting of a 1/8-inch diameter, 6-foot lead column of 5A molecular sieve, followed by a 1/8-inch diameter, 12-foot-long Haysep-Q™ column. The He carrier flow was set at 20mlmin^−1^. The SF_6_ peak was registered on a thermal conductivity detector and then isolated by freezing into a liquid-nitrogen-cooled trap. The isotope composition of the purified SF_6_ was determined by dual-inlet gas-source mass spectrometry monitoring ion beams at *m/e* of 127, 128, 129 and 131, using a Thermo Finnigan MAT 253 gas source mass spectrometer at CAS Key Laboratory in Hefei^42^. Sulphur isotope data are presented using delta notation δ^*x*^S = [(^*x*^S/^32^S)_sample_/(^*x*^S/^32^S)_reference_ − 1], where *x* is 33, 34 or 36; and capital delta notation ∆^33^S = [(^33^S/^32^S)_sample_/(^33^S/^32^S)_reference_ − ((^34^S/^32^S)_sample_/(^34^S/^32^S)_reference_)^0.515^] and ∆^36^S = [(^36^S/^32^S)_sample_/(^36^S/^32^S)_reference_ − ((^34^S/^32^S)_sample_/(^34^S/^32^S)_reference_)^1.9^]. Capital delta and delta values are given in units of per mil (‰).One-sigma uncertainties on mass-dependent reference materials are better than ± 0.2‰, ± 0.01‰ and ± 0.2‰ in δ^34^S, ∆^33^S and ∆^36^S, respectively. Uncertainties on the measurements reported here are estimated to be better than ± 0.2‰, ± 0.01‰ and ± 0.2‰.

## Supplementary Information

Supplementary Information.
