# Supplementary material for: Submarine metalliferous carbonate mounds in the Cambrian of the Baltoscandian Basin induced by vent networks and water column stratification
Source: Sci Rep. 2022 May 19;12:8475. doi: 10.1038/s41598-022-12379-y (PMC9119982; doi:10.1038/s41598-022-12379-y)
Supplement: Supplementary file 1 — Supplementary Information. [file 41598_2022_12379_MOESM1_ESM.docx]

**REPOSITORY DATA**

**Figure 1.** Stratigraphic subdivision and completeness of the Alum Shale Formation at the Bruddesta-Äleklinta and Degerhamn sections of Öland and the δ^13^C_org_ curve obtained from the Cambrian and Lower Ordovician (Tremadocian) Alum Shale Formation of the Grönhögen-2015 drill core^1^. Biostratigraphic data after^1-4^. The Cambrian lithostratigraphic succession of Estonia and Ingria (Russia) is updated: 1, Exporrecta Conglomerate Bed; 2, Kakeled Limestone Bed; 3, limestones; 4, black shales; 5, non-deposition intervals; 6, sandstones; 7, stratigraphic position of glendonites and mound crusts described in this work; 8, massive glendonite accumulations described in ^5^.


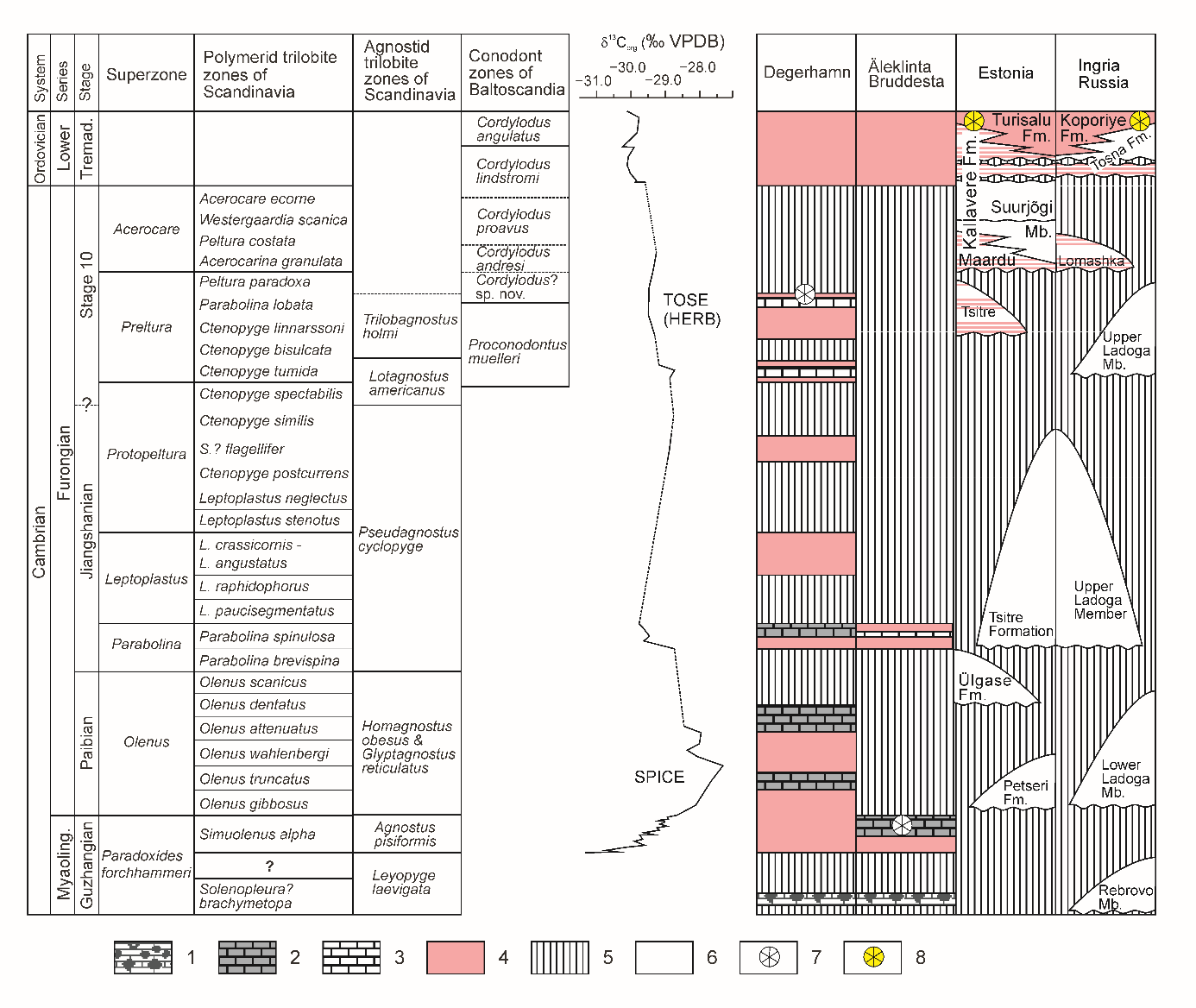


**Figure 2.** Raman spectra of carbonate crusts and relative wavenumbers of calcite, vaterite and Ca-O^6-8^.


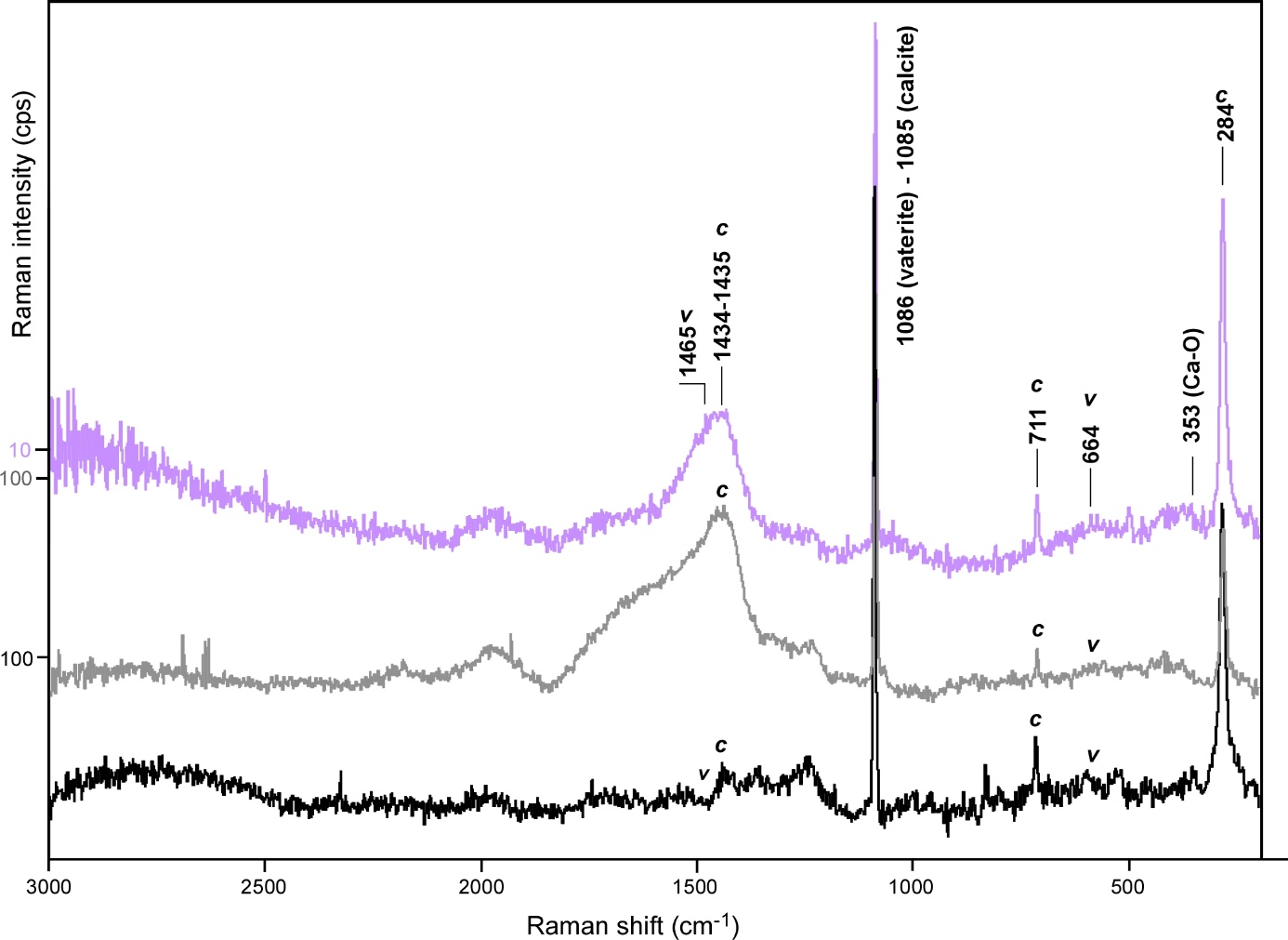


**Figure 3. A.** Cloudy calcite (*ca1*) with cements of clean calcite (*ca2*) under plane polarized light. **B.** Primary solid inclusions (red arrow) and fluid inclusions (yellow arrow) in *ca2*. Fluid inclusions are mostly all-liquid at room temperature, under plane polarized light. **C‒E.** Details of all-liquid primary fluid inclusions in *ca2*, under plane polarized light. D and E correspond to FIA 9 (see Table 2); bubble arrowed. The lines join same FI in the two photos.

**
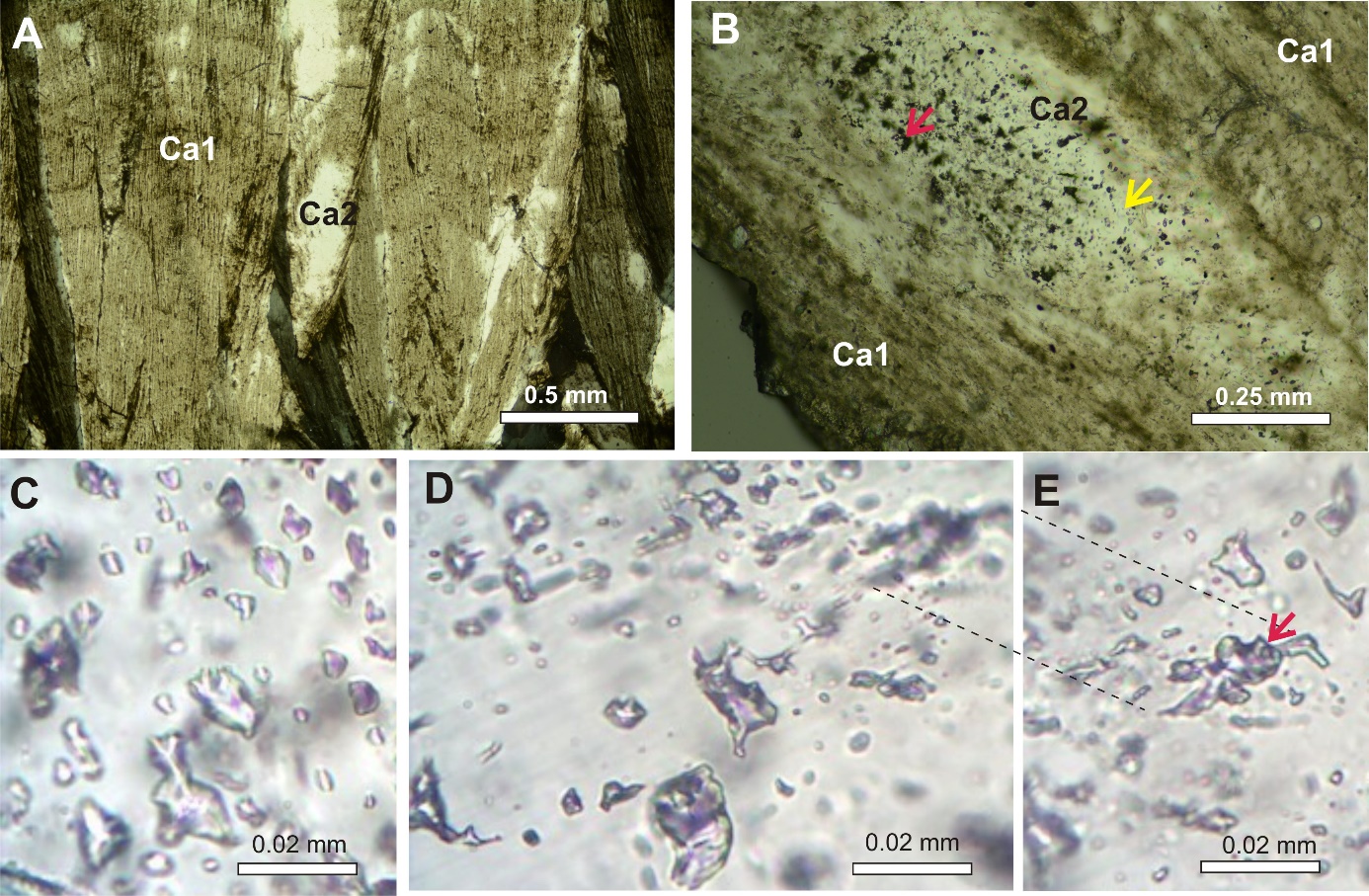
**

**Figure 4.** Final melting of ice temperatures (*T*_mice_) and homogenization temperatures (*T*_h_) histograms for primary fluid inclusions in dirty calcite (*ca1*), clean calcite (*ca2*) and fracture infills (*ca3*). Note that the *T*_mice_ and *T*_h_ of *ca1* and *ca2* fluid inclusions are similar. In *ca3* the *T*_h_ have not been measured because they would be meaningless (see text).

**
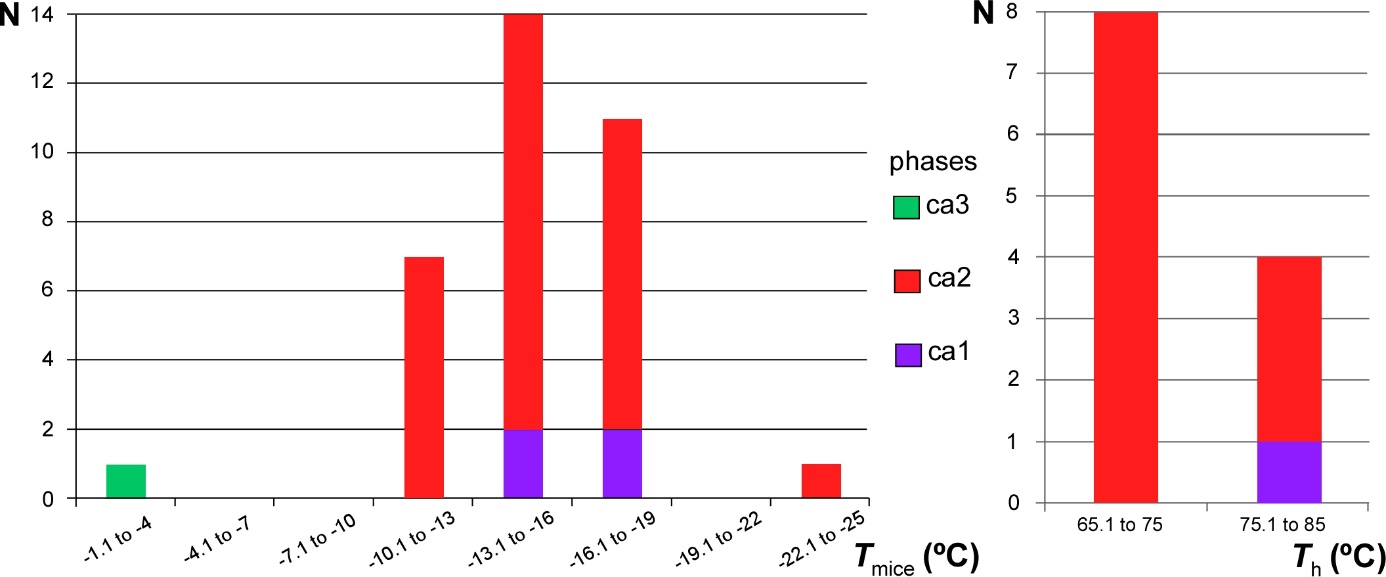
**

**Figure 5.** **A.** Fracture filling (*ca3*) crosscutting *ca1* crystals. Primary fluid inclusions in *ca3* are encircled and are very rich in gas, under plane polarized light. **B.** Detail of a fluid inclusion very rich in gas in fracture filling, under plane polarized light. The arrow is pointing to the meniscus (boundary liquid-gas). **C.** Detail of a biphasic fluid inclusion (1, encircled) together with an all-gas fluid inclusion (2, shown outside because the two FI are in planes slightly different). The different liquid to vapor ratios among the FIs indicates heterogeneous entrapment. FIA 1.

**
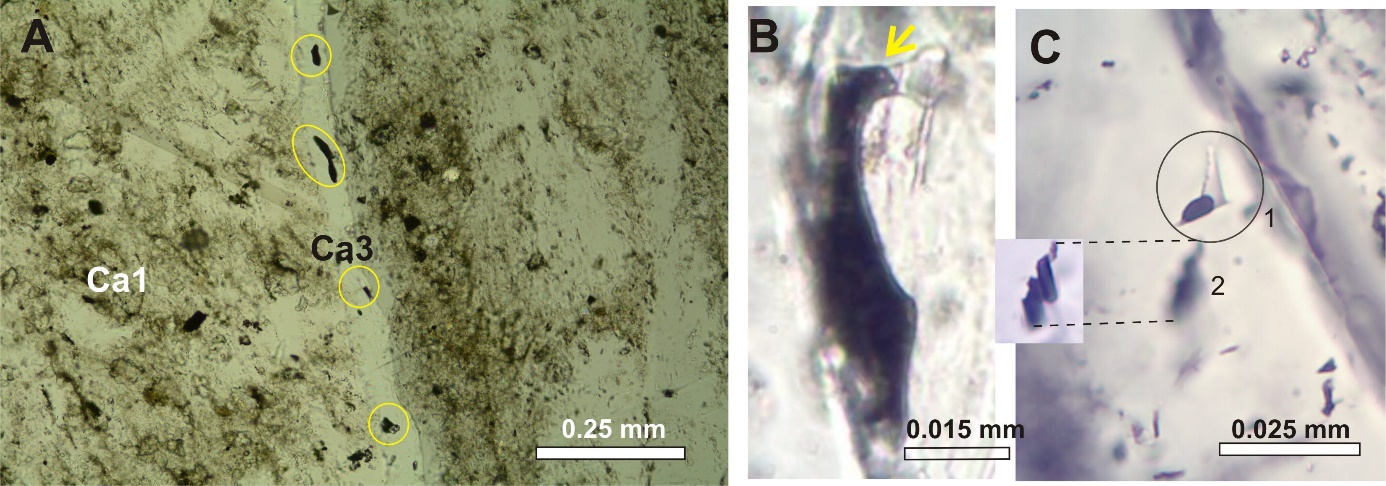
**

**Table 1.** Carbon and oxygen isotope data from the Cambrian mound crusts and glendonitic aggregates at Bruddesta-Äleklinta and Degerhamn, Öland.

**Table 2.** Microthermometric measurements on fluid inclusions primary to dirty calcite (Ca1), to clean calcite (Ca2) and to fracture fillings (Ca3). Sample names are is indicated as GRU, HORN and ALEK. FI: number of the FI. Size in µm. Liquid %: proportion of liquid at room temperature. FIA: Fluid Inclusion Assemblage. Th: Homogenization temperature. Tn: Nucleation temperature. Tmice: Final melting temperature of ice. Tfm: Temperature of first melting; all temperatures in ºC. NV: non visible. Decrep.: FI partially decrepitated.

**Table 3.** Sulphur isotope data from the Cambrian sulphide clasts encased in the metalliferous carbonate mounds at Bruddesta, Öland; AVS = acid volatile sulphides, BRU = Bruddesta and CRS = chromium-reducible sulphides.

**References**

1. Ahlberg, P. *et al*. Integrated Cambrian biostratigraphy and carbon isotope chemostratigraphy of the Grönhögen-2015 drill core, Öland, Sweden. *Geol. Mag*. **156**, 935–949 (2019).

2. Bagnoli, G. & Stouge, S.. Upper Furongian (Cambrian) conodonts from the Degerhamn quarry road section, southern Öland, Sweden. *GFF* **136**, 436–458 (2014).

3. Rasmussen, B. W., Rasmussen, J. A. & Nielsen, A. T. Biostratigraphy of the Furongian (upper Cambrian) Alum Shale Formation at Degerhamn, Öland, Sweden. *GFF* **139**, 92–118 (2017).

4. Nielsen, A. T., Høyberget, M. & Ahlberg, P. The Furongian (upper Cambrian) Alum Shale of Scandinavia: revision of zonation. *Lethaia* **53**, 462–485 (2020).

5. Popov, L. E. *et al*. 2019. Glendonite occurrences in the Tremadocian of Baltica: first Early Palaeozoic evidence of massive ikaite precipitation at temperate latitudes. *Sci. Rep*. **9**, 7205 (2019).

6. Larkin, P. J. IR and Raman Spectroscopy. Principles and Spectral Interpretation (Elsevier, Amsterdam, 2011).

7. Frezzotti, M. L., Terce, F. & Casagli, A. Raman spectroscopy for fluid inclusion analysis. *J. Geochem. Explor*. **112**, 1–20 (2012).

8. Sánchez-Pastor, N. *et al*. Crystallization of ikaite and its pseudomorphic transformation into calcite: Raman spectroscopy evidence. *Geochim. Cosmochim. Acta* **175**, 271–281 (2016).
